# The effect of obesity and subsequent weight reduction on cardiac morphology and function in cats

**DOI:** 10.1186/s12917-024-04011-0

**Published:** 2024-04-24

**Authors:** Catheryn Partington, Hannah Hodgkiss-Geere, Georgia R. T. Woods, Joanna Dukes-McEwan, John Flanagan, Vincent Biourge, Alexander J. German

**Affiliations:** 1 Institute of Infection, Veterinary, Ecological and Sciences, Department of Small Animal Clinical Sciences, Teaching Hospital, University of Liverpool, Neston, UK; 2 Institute of Life Course and Medical Sciences, Department of Small Animal Clinical Sciences, Teaching Hospital, University of Liverpool, Neston, UK; 3 Royal Canin Research Center, Aimargues, France; 4 Present address: Department of Veterinary Medicine, University of Cambridge, Madingley Road, Cambridge, CB3 0ES UK

**Keywords:** Cardiac biomarkers, Diastolic function, Echocardiography, Hypertrophic cardiomyopathy

## Abstract

**Background:**

In people, obesity is a risk factor for cardiovascular disease, associated with systemic hypertension, cardiac remodelling and systolic and diastolic dysfunction. Weight reduction can reverse myocardial remodelling and reduce risk of subsequent cardiovascular disease. In cats, far less is known regarding the effects of obesity and subsequent weight reduction on cardiovascular morphology and function. This prospective study aimed to assess cardiac morphology and function, heart rate variability, cardiac biomarkers and body composition before and after controlled weight reduction in cats with obesity. Body composition analysis (by dual energy x-ray absorptiometry, DEXA) and cardiovascular assessment (echocardiography, systemic arterial systolic blood pressure, electrocardiography, plasma cardiac biomarkers) were performed prior to weight management in twenty cats with obesity. These investigations were repeated in eleven cats that reached target weight.

**Results:**

At baseline, systemic hypertension was not documented, but the majority of cats with obesity (15 out of 19) showed echocardiographic evidence of diastolic dysfunction. Eleven of 20 cats had increased maximal end-diastolic septal or left ventricular free wall thickness (≥ 6.0 mm) at baseline. Median (interquartile range) percentage of weight lost in the cats reaching target weight was 26% (17–29%), with a median reduction in body fat mass of 45% (26–64%). Both the end-diastolic left ventricular free wall (median magnitude of change -0.85 mm, IQR -0.05 mm to -1.55 mm, *P* = 0.019; median percentage reduction 14.0%) and end-diastolic interventricular septum (median magnitude of change -0.5 mm, IQR -0.2 mm to -1.225 mm, *P* = 0.047; median percentage reduction 7.9%) thickness decreased after weight reduction. Following weight reduction, pulsed wave tissue Doppler imaging of the left ventricular free wall was consistent with improved diastolic function in 4 out of 8 cats, however there was no significant difference in overall diastolic function class. Further, there was no change in heart rate variability or cardiac biomarkers with weight reduction.

**Conclusion:**

An increase in left ventricular wall thickness and diastolic dysfunction were common echocardiographic features in cats with obesity within our study and may be reversible with successful weight and fat mass loss. Further studies are required to clarify the clinical consequences of these findings.

**Supplementary Information:**

The online version contains supplementary material available at 10.1186/s12917-024-04011-0.

## Introduction

The cardiovascular effects of obesity in people are well documented, not only resulting from the association with atherosclerosis and ischaemic myocardial disease but also from the effects on cardiac morphology and function [1, 2]. Obesity is associated with chronic increases in preload and afterload, activation of the renin–angiotensin–aldosterone system and sympathetic nervous system as well as alterations in cellular homeostasis and energy metabolism. One consequence is that systemic hypertension is common amongst people with obesity [2]. Left ventricular (LV) hypertrophy is commonly reported, with or without concurrent chamber dilatation [3]. Impaired diastolic and systolic function are also frequently seen [2]. Consequently, obesity is an independent cardiovascular risk factor in people [2].

Obesity in pet cats and dogs is also a major health concern, associated with increased morbidity and mortality [4, 5]. The cardiovascular effects of obesity reported in dogs share some similarities to those observed in people, although findings amongst reports are variable [6–9]. Varying patterns of left ventricular hypertrophy are reported in dogs with obesity [6, 7], in addition to diastolic dysfunction in some [6, 8], but not all [7] reports, whilst the presence of systemic hypertension in dogs with obesity remains variable between studies [6–9]. In cats, less is known regarding the cardiovascular effects of obesity. Litster and Buchanan [10] reported no significant echocardiographic changes in cats with obesity compared with cats in ideal weight, besides an increase in precordial distance and increased thickness of the right ventricular free wall. In contrast, De Souza et al. [11] reported an increase in vertebral heart score, diastolic left ventricular free wall (LVFWd) thickness and blood pressure in 20 cats with obesity, whilst Stepien et al. [12] similarly reported increased wall thickness but reported no significant change in blood pressure. Diastolic function was not assessed in either report; however, Champion [13] reported an association between increased left ventricular wall thickness (LVWT) and the presence of diastolic dysfunction in 22 cats that were overweight or had obesity.

Hypertrophic cardiomyopathy (HCM) is the most common acquired heart disease of cats, effecting around 14.7% of the population, and is defined as increased LVWT in the absence of abnormal loading conditions [14]. Cats with a greater bodyweight are reported to have an increased risk of developing HCM, whilst the association between body condition score (BCS) and risk of developing HCM varies between reports [14, 15]. Further, other disease processes may result in increased LVWT, mimicking a HCM phenotype. Thus, uncertainty about the impact of obesity on cardiac structure and function may hinder interpretation of increased LVWT and thus complicate the diagnosis of HCM in cats with obesity.

In humans, obesity is also associated with an increase in heart rate, mediated for the most part by altered sympathovagal balance [2]. Heart rate variability (HRV) is an indicator of this autonomic tone and has been used to predict risk of cardiovascular disease in people with obesity [16]. Studies assessing HRV in dogs with obesity show varied results. While Champion [13] reported no difference in heart rate variability in cats in overweight or obese condition, compared with those in ideal body condition score; however, to the authors’ knowledge, the effect of controlled weight reduction in cats has not been assessed.

Therefore, the first aim of the current study was to assess a cohort of cats with obesity for the presence of systolic or diastolic dysfunction, altered wall thickness and systemic hypertension. A second aim was to monitor for changes in echocardiographic variables, cardiac biomarkers, and systemic blood pressure in response to a controlled weight reduction programme, using dual energy x-ray absorptiometry (DEXA) to quantify changes in body composition. Thirdly, we aimed to monitor for changes in autonomic balance by examining changes in HRV during controlled weight reduction. We hypothesised that cats with obesity would show signs of diastolic dysfunction and increased left ventricular wall thickness, which may improve with weight reduction.

## Results

### Study animals

Twenty cats were enrolled, of which 11 reached their weight reduction target. All cats were of the domestic shorthair breed, with a median age of 7.4 years (interquartile range [IQR] 5.6 -8.9 years) at time of enrolment (Supplementary Table 1). There were 8 females and 12 males (all neutered). Median weight for all cats at enrolment was 7.2 kg (IQR 6.46- 8.38 kg) with a median body condition score (BCS) of 8/9 (IQR 8–9). Two cats did not undergo DEXA post-weight reduction due to lack of consent for sedation. There was no difference in the age (*P* = 0.518, *r [effect size]* = 0.14), bodyweight (*P* = 0.820, *r* = 0.05) or BCS (*P* = 0.222, *r* = 0.27) at time of inclusion between those cats that did and did not achieve target bodyweight.

### Baseline cardiovascular variables (all cats)

The baseline data for cardiovascular variables for all cats is shown in Supplementary Tables 1 and 2. Baseline systolic blood pressure (SBP) was within the reference interval [RI] of < 160 mmHg (median 135 mmHg, IQR 120 – 147 mmHg) in all cats, except the two for which this data were missing (due to measurement not being tolerated). Both N-terminal Pro B-type natriuretic peptide (NT-proBNP) and high-sensitivity cardiac troponin I (hs-cTnI) were missing for three cats at inclusion. Concentration of NT-proBNP was less than the laboratory upper reference range of 100 pmol/L in 17/17 cats (median 23.9 pmol/L, IQR 23.9–27.5 pmol/L). Of the two cats that had increased hs-cTnI, the concentration was only mildly increased (0.055 ng/mL, RI ≤ 0.04 ng/mL) in one; in this cat, NT-proBNP was 61.0 pmol/L and left atrial (LA) size was normal, but LVWT was mildly increased (diastolic interventricular septum [IVSd] 6.0 mm). In the second cat, hs-cTnI was moderately increased (0.134 ng/mL); in this cat, NT-proBNP was 31.0 pmol/L, LA size was normal but there was a mild increase in LVWT (IVSd 6.3 mm). In both cats, electrocardiography (ECG) revealed normal sinus rhythm. Normal hs-cTnI concentrations were documented at baseline in all other cats (median 0.007 ng/mL, IQR 0.004–0.038 ng/mL). Median vasovagal tonus index (VVTI) was 5.21 (IQR 4.93–5.70).

On echocardiography, LVWT was increased (defined as ≥ 6.0 mm) in most (11/20) cats, with wall thickness being equivocal (defined as 5.5—5.9 mm) in an additional 5/20 cats. The increase in LVWT affected the interventricular septum more often than the free wall, with an IVSd ≥ 6.0 mm in 11/20 cats and an IVSd of 5.5- 5.9 mm in 2/20 (6.05, IQR 5.23–6.58 mm), compared with a LVFWd ≥ 6.0 mm in 5/20 cats and 5.5- 5.9 mm in 7/20 cats (5.55, IQR 5.13–5.98 mm). One cat had systolic anterior motion of the mitral valve, causing mild dynamic left ventricular outflow tract (LVOT) obstruction (LVOT velocity 2.25 m/s). A second cat had mild chordal anterior motion, but no obvious increase in LVOT velocity (1.34 m/s). Most cats had no LA dilation; short axis left atrium to aorta ratio (LA/Ao) < 1.6 in 19/20 cats (1.30; IQR 1.22–1.44) and maximal left atrial diameter (LADmax) < 16 mm in 12/18 cats (15.05 mm, IQR 13.70–16.03 mm).

For one cat, diastolic function class was not determined due to incomplete echocardiographic data. At baseline, diastolic function was considered to be normal in 4/19 cats, whilst 9/19 cats had impaired relaxation and 6/19 had pseudonormal diastolic function. Tissue Doppler imaging of the LVFW was consistent with impaired relaxation (E’/A’ < 1) in 13/17 cats (pulsed wave tissue Doppler imaging [pw-TDI] data incomplete for 2 cats). Transmitral flow showed an impaired relaxation pattern in 7/17 cats and isovolumetric relaxation time (IVRT) was increased in 7/19 cats (median 59.0, IQR 54.0–63.0 ms, RI < 60.0 ms). For the right lateral wall E’/A’ was < 1 in 18/18 cats (pw-TDI of the right wall data incomplete for 2 cats). There was no evidence of systolic dysfunction based on fractional shortening (54%, IQR 48.3–59.5%, RI > 30%) in any cat; however, pw-TDI S’ velocities of the septal and lateral walls were mildly decreased in 8/19 and 9/19 cats, respectively (IVS 6.5 cm/s, IQR 4.9–9.0 cm/s; LVFW 6.6 cm/s, IQR 4.8–9.4 cm/s; RI > 6 cm/s [17], for the 19 cats).

Six-lead ECG in 19/20 cats showed sinus rhythm, one of which had a left anterior fascicular block pattern and two showed evidence of intraventricular conduction disturbance (as shown by altered QRS morphology). One cat had sinus bradycardia with a heart rate of 120 bpm. The median heart rate for all cats was 180 bpm (IQR 160–215 bpm).

### Weight reduction outcomes

Outcomes of weight reduction, for the 11 cats that reached their target weight, are shown in Table 1. The median duration of weight loss was 190 days (IQR 162–229 days) and median weight lost was 1.45 kg (IQR 1.16–2.35 kg), equating to a decrease of 26% (IQR 17–29%) of starting body weight. Median rate of weight loss was 1.64% per week (IQR 1.07–2.29%). Body condition score decreased by a median of 3 units (IQR 3–3, *P* = 0.003, *r* = 0.90), lean mass change was -7.6% (IQR -3.4% to -8.4%; *P* = 0.008, *r* = 0.89) and body fat change was -45% (IQR -26% to -64%, *P* = 0.008, *r* = 0.89).

**Table 1:** Weight reduction and dual energy x-ray absorptiometry (DEXA) data for the cats that achieved target weight reduction

**Variable**	**Number of cats**	**Before weight reduction**		**After weight reduction**		***P* value**	***r value***^a^ *[Effect size]*
		**Median**	**(IQR)**	**Median**	**(IQR)**		
**Weight (kg)**	11	7.20	6.40–9.05	5.36	5.18–6.04	0.003	0.88
**BCS (/9)**	11	8	8–9	5	5–7	0.003	0.90
**Body fat (%)**	9	35.1	29.5–48.3	17.4	10.9–31.4	0.008	0.89
**Body fat (g)**	9	2138.0	1792.0–3495.0	976.0	481.5–1540.5	0.008	0.89
**Lean mass (g)**	9	4095.0	3469.0–4707.0	3547.0	3133.5–4316.0	0.008	0.89
**BMC (g)**	9	143.9	127.0–168.1	125.1	113.4–145.4	0.008	0.89

Eleven cats achieved target weight loss, for which repeat DEXA was available in 9

*BCS* Body condition score, *BMC* Bone mineral content, *IQR* Interquartile range

^a^Effect size was calculated from the z statistic, and used to calculate r, as described by Cohen [18, 19], using the formula r = Z /√N (where Z is the z statistic and N is the sample size). The effect size, r, was interpreted according to the rules of Cohen [19], whereby values of 0.1, 0.3 and ≥ 0.5 were considered small, medium and large effects respectively [18, 19]

### Changes in cardiovascular variables with weight reduction

Cardiovascular variables before and after weight reduction are shown in Table 2. Systolic blood pressure measurement was missing for one cat after weight reduction. Two cats were hypertensive on this second assessment (SBP 170 mmHg and 180 mmHg), whilst all the remaining cats were normotensive. There was no change in SBP (*P* = 0.438, *r* = 0.26), heart rate (*P* = 0.932, *r* = 0.26) or HRV (*P* = 0.790, *r* = 0.08) with weight reduction. Cardiac biomarkers were missing for four cats after weight reduction; for the remaining seven cats, there was no change in NT-proBNP (*P* = 0.144, *r* = 0.55) or hs-cTnI (*P* = 0.461, *r* = 0.28) concentrations. Of the two cats with increased hs-cTnI at baseline, this normalised after weight reduction in one cat, but remained mildly increased in the other (0.045 nmol/L, RI ≤ 0.04); NT-proBNP concentration was increased (110 pmol/L, RI < 100) and an increase in LVWT was also seen in this cat. For all other cats, cardiac biomarkers remained within normal reference range at this second time point.

**Table 2:** Cardiovascular variables pre- and post- weight reduction for the cats that achieved target weight reduction

Variable	Number of cats	Before weight reduction		After weight reduction		*P* value	*r* value
		**Median**	(IQR)	**Median**	(IQR)		
**ECG heart rate (bpm)**	11	180	160–200	180	160–200	0.932	0.26
**Heart rate variability (VVTI)**	11	5.24	4.96–5.72	4.87	4.56–6.00	0.790	0.08
**SBP (mmHg)**	10	134	116–150	129	120–157	0.438	0.26
**Hs-cTnI (ng/mL)**	7	0.007	0.004–0.040	0.005	0.004–0.011	0.461	0.28
**NT-proBNP (pmol/L)**	7	24.0	23.9–31.0	23.9	23.9–75.0	0.144	0.55
**LA/Ao**	10	1.3	1.26–1.46	1.41	1.27–1.57	0.386	0.26
**LADmax (mm)**	10	15.6	14.4–16.1	15.5	14.2–16.3	0.646	0.14
**Max-IVSd (mm)**	11	6.3	5.8–6.6	5.9	5.5–6.8	0.142	0.35
**Max-IVSd (mm) excluding 1 HCM case**	10	6.4	5.7–6.8	5.9	5.2–6.7	**0.047**	0.63
**Max-LVFWd (mm)**	11	5.5	5.1–6.0	5.3	4.9–5.8	**0.045**	0.60
**Max-LVFWd (mm) excluding 1 HCM case**	10	5.7	5.2–6.4	5.3	4.6–5.8	**0.019**	0.74
**E velocity (m/s)**	10	0.64	0.58–0.70	0.64	0.49–0.70	0.083	0.52
**MV E/A**	6	1.01	0.85–1.24	1.02	0.88–1.29	0.309	0.38
**IVRT (ms)**	10	59.0	53.0–63.0	56.0	51.0–62.0	0.859	0.05
**Septal (IVS) E’/A’**	9	0.60	0.55–0.82	0.75	0.58–0.98	0.085	0.57
**Lateral (LVFW) E’/A’**	9	0.86	0.61–1.34	1.2	0.77–1.53	**0.012**	0.89
**Right E’/A’**	9	0.86	0.56–0.78	0.66	0.52–0.80	0.766	0.10
**Septal (IVS) S’ (cm/s)**	10	7.15	4.83–9.33	7.4	5.60–8.40	0.646	0.15
**Lateral (LVFW) S’ (cm/s)**	10	7.20	5.53–10.03	6.50	5.20–7.80	0.139	0.47
**Fractional shortening (%)**	10	53.0	44.0–61.0	50.0	40.0–53.0	0.182	0.40

The *r* value reflects effect size

*E’/A’* pulsed wave tissue Doppler imaging derived diastolic myocardial E and A wave velocity ratio, *ECG* Electrocardiography, *hs-cTnI* High sensitivity cardiac troponin I, *IQR* Interquartile range, *IVRT* isovolumetric relaxation time, *IVSd* end-diastolic interventricular septum thickness, *LA/Ao* left atrium to aorta ratio, *LADmax* Maximal left atrial diameter, *LVFWd* End-diastolic left ventricular free wall thickness, *MV E/A* Mitral valve E and A wave velocity ratio, *NT-proBNP* N-type N-terminal pro-brain natriuretic peptide, *S’* Pulsed wave tissue Doppler imaging derived myocardial systolic velocity, *SBP* Systolic blood pressure, *VVTI* Vasovagal tonus index

**Fig. 1:**
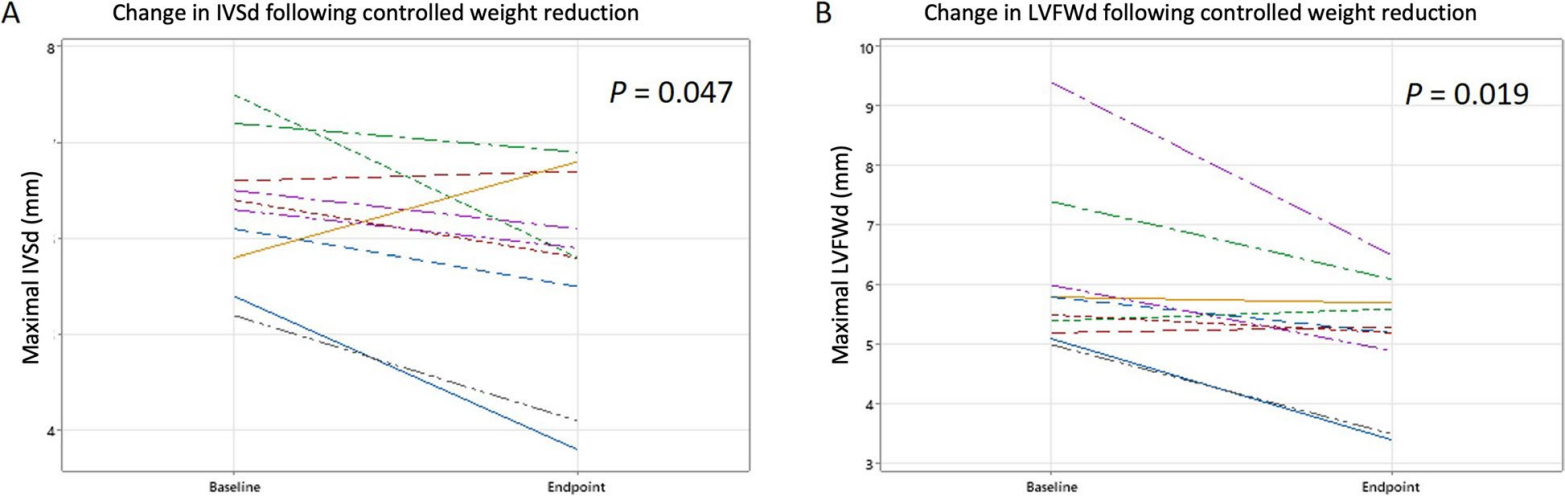
Changes in left ventricular wall thickness following controlled weight reduction in 10 cats. Line plots showing the changes in **a** maximal end-diastolic interventricular thickness and **b** maximal end-diastolic left ventricular free wall thickness with weight reduction for each cat achieving target weight reduction. The cat with progressive HCM has been excluded. IVSd: end-diastolic interventricular septum thickness, LVFWd: end-diastolic left ventricular free wall thickness

After weight reduction, 4/11 cats had equivocal LVWT and five increased LVWT (compared with 1/11 and 8/11 respectively, at baseline). Six-lead ECG showed sinus rhythm in all cats; in addition, one cat had a left anterior fascicular block pattern and one had evidence of intraventricular conduction disturbance based on QRS morphology. In one cat, there was progression of LVWT and increased cardiac biomarkers (SBP was within the RI); this cat was considered to have progressive preclinical HCM and was not included in further statistical analysis. In the remaining 10 cats, there were decreases in both the maximal LVFWd (median magnitude of change -0.85 mm, IQR -0.05 mm to -1.55 mm, *P* = 0.019, *r* = 0.74; median percentage reduction 14.0%) and maximal IVSd (median magnitude of change -0.5 mm, IQR -0.2 mm to -1.225 mm, *P* = 0.047, *r* = 0.63; median percentage reduction 7.9%) after weight reduction (Fig. 1). There was no change in any other two-dimensional (2D) echocardiographic variable with weight reduction. There was a trend for the diastolic function class to improve, but this did not reach statistical significance (*P* = 0.763). When assessing individual variables of diastolic function, there was an increase in the LVFW pw-TDI derived E’A’ ratio with weight reduction (*P* = 0.012, r = 0.89); this ratio normalised in 4/8 cats, remained normal in 2/8 and remained consistent with impaired relaxation in 2/8 cats. There were no changes in the other variables of diastolic function, nor of systolic function, with weight reduction.

## Discussion

In people, the cardiovascular effects of obesity are well documented [2]; however, less is known about the cardiovascular effects of obesity in cats. In the current study, cats with obesity frequently had an increase in LVWT and diastolic dysfunction, with some improvement following weight reduction suggesting that these changes might be reversible. DEXA body composition results confirmed loss of fat mass as expected during weight reduction.

Fifty-five percent (11/20) of cats in this study had at least one wall measurement equal to or exceeding 6 mm (IVSd ≥ 6 mm in 11/20; LVFWd ≥ 6 mm in 5/20), consistent with a HCM phenotype [20]. This corresponds to the changes seen in people and dogs with obesity in which LV hypertrophy is commonly reported [2, 7–9]. Our findings are also consistent with those of Stepien et al. [12] who reported LV hypertrophy in overweight cats. Development of increased LVWT in obesity is likely to be multifactorial involving both myocardial remodelling in response to altered preload and afterload, as well as myocardial and epicardial lipid deposition and altered cellular metabolism [1, 21, 22]. Insulin resistance and increased serum insulin-like growth factor concentration have been identified in some cats with HCM, supporting this role of metabolic changes in the development of increased LVWT [23, 24]. Further, an association between both bodyweight and BCS and serum concentrations of insulin, insulin-like growth factor and glucose has been shown in cats with subclinical HCM [23]. The presence of increased LVWT in cats with obesity brings into question the possible impact on cardiac function and long-term cardiac morbidity and mortality, as well as highlighting the need for clarifying interpretation of LVWT measurements in cats with obesity. Häggström et al. [25] showed that many echocardiographic variables (including LA size and LVWT) increase with increasing body weight and, therefore, proposed the use of allometric scaling to bodyweight. However, since the BCS of cats in that study were not documented, it was unclear as to whether the increase in cardiac dimensions noted was associated with lean mass, fat mass or both. Most cats in our study had normal LA size, despite above average bodyweight and increased LVWT; this might suggest that increased fat mass and increased lean mass do not have the same effects on cardiac dimensions.

Decreased LVWT with weight reduction is seen in both people and dogs with obesity [7, 9, 26], and was also seen in this study, with a significant decrease in both the LVFWd and IVSd (median reduction of -0.85 mm and -0.5 mm for the LVFWd and IVSd respectively; following exclusion of one cat deemed at follow-up to have primary HCM). In people, improvement in LVWT with weight reduction is in-part a result of reduced afterload secondary to improved systemic hypertension [2, 22]. However, systemic hypertension was excluded in cats from the current study, indicating this reverse remodelling must be multifactorial; possible mechanisms could include a decrease in lipid accumulation within cardiomyocytes and metabolic changes [1, 22]. In one study, there was a decrease in LVWT in cats with subclinical HCM following diet change (starch restricted, high protein diet supplemented with eicosapentaenoic acid and docosahexaenoic acid), despite no clinically significant change in body weight or BCS [27]. In the current study, the change in diet may also have influenced LVWT and, therefore, we cannot definitively conclude that the decreased wall thickness was a result of reduced body fat.

Diastolic dysfunction was evident in many of the cats with obesity in the current study, but systolic dysfunction was not observed. Both diastolic and systolic dysfunction are reported in people with obesity, resulting in an increased risk of heart failure [2, 21], whilst diastolic dysfunction has also been reported in dogs with obesity [6]. In our study, although the change in overall diastolic function class did not change with weight reduction, there was a significant change in the pw-TDI E’A’ ratio of the LVFW, which normalised in 4/6 cats (impaired relaxation pre-weight reduction). This suggests an improvement in diastolic function following controlled weight reduction, as reported in humans [28, 29]; in contrast, no such improvement was seen in dogs [30]. It would be necessary to study a larger number of cats in order to assess this further. Development of diastolic dysfunction is multifactorial: triglyceride accumulation increases apoptosis of cardiomyocytes, renin–angiotensin–aldosterone system activation and increased aldosterone concentration contribute to myocardial fibrosis, whilst inflammatory cytokines contribute to fibrosis and increased wall stiffness, all contributing to diastolic dysfunction [2]. However, many other variables including age and SBP may affect diastolic function, with diastolic dysfunction being a ‘normal’ finding on echocardiography of older animals. Given the small sample size, we did not attempt to correct for these potentially confounding variables in the statistical comparisons. However, our cohort did include young cats in which diastolic dysfunction would not be expected (age range 1.3 – 12.8 years); furthermore, improvement in diastolic function would not be seen if a result of age, we thus concluded that obesity was a more likely cause.

Given that obesity is associated with increased sympathetic drive [31], we hypothesised that cats with obesity would have a decrease in HRV (an indicator of sympathetic tone), which may improve following controlled weight reduction. However, there were no changes in either heart rate or HRV in the current study. This is likely to reflect the increased sympathetic drive typically seen in cats attending veterinary clinics, which makes it difficult to assess possible effects of obesity on autonomic tone. Use of ambulatory ECG may be a better way to assess heart rate and HRV, although even this approach might be hampered by stress and subsequent increased sympathetic tone. Further, frequency domain analysis of HRV (such as by Holter ECG), instead of the time domain method VVTI, may give different results. Although, in one Holter study, there was no difference between cats that were either overweight or had obesity, compared with cats in ideal body condition [13].

Cardiac biomarkers were within reference range for all but two cats in the current study, with no significant change following weight reduction. This might suggest that obesity had not contributed to clinically-significant increases in wall stress or myocardial injury, despite the increase in wall thickness. However, in human heart failure patients with obesity, a smaller increase in NT-proBNP occurs compared with those with a normal body mass index [32] suggesting a more complex interaction between NT-proBNP and obesity. In the one cat with persistently increased hs-cTnI, there was an increase in NT-proBNP, LA size and LVWT at follow up despite weight reduction. This cat was diagnosed with primary HCM.

Obesity-related cardiac dysfunction is associated with increased morbidity and an increased risk of cardiovascular death in people. Considering the worldwide prevalence of both obesity and HCM in the pet cat population, it is important to develop a better understanding of the effects of obesity on feline cardiac structure and function. There is an obesity paradox in human cardiology, whereby patients with obesity have longer survival times once heart failure has developed [33, 34] however, human patients with HCM and obesity have more advanced echocardiographic changes, worse heart failure scores and are more symptomatic, than those of ideal bodyweight [34–36]. These findings suggest that obesity may be an important modifier of HCM penetrance, severity and progression [37]. A similar obesity paradox has been reported in cats with heart failure in one study [38] although no association between bodyweight and prognosis was seen in a second study of cats [39] emphasising the complexity of the relationship between obesity and heart disease. Although we did not examine effects of obesity on cats with HCM in the current study, our results did demonstrate that obesity is associated with increased LVWT and diastolic dysfunction, which could be mistaken for, or mask, feline HCM. Further research is required to understand whether obesity should be considered as a HCM phenocopy, or whether obesity acts as a modifier of HCM penetrance and expression in cats.

The main limitation of the current study is the small number of cats; the study might have been underpowered as a result, meaning that subtle changes in cardiac function might have been missed. A power calculation to determine the required study population size was not performed; instead, the number of cats recruited was instead pragmatic, based on the number of cases likely to be recruited over the study time frame. However, our population size was equivalent to those used in recent canine studies [6, 7, 9]. Given concerns that the study might have been underpowered, the *P* value was not adjusted for multiple comparisons, which is itself a limitation. As a third study limitation, when comparing echocardiographic findings before and after weight reduction, few echocardiographic differences were identified. This made it inappropriate to explore associations between changes in body composition (based on DEXA) and changes in echocardiographic variables. Further, many of the cardiovascular measurements might have been affected by confounding variables; for example, diastolic function is affected by age, a variable which was not controlled in this study. However, a wide age range was present in the study cats, thereby making age-effects less likely. Heart rate can affect some echocardiographic variables and might also have been a confounder when comparing pre and post weight reduction data; however, such an impact should be minimal as there was no significant change in heart rate.

One challenge of this study, and another inherent limitation, was the difficulty in interpreting the cause of increased LVWT in study cats. Considering the prevalence of subclinical HCM in the feline population of around 14.7% [14], some cats with increased LVWT at baseline might have had subclinical HCM. However, if this were the sole cause of hypertrophy, this would not be expected to improve with time and thus a reduction in wall thickness would not be expected after weight reduction. The possibility of concomitant subclinical HCM is more likely to have underestimated changes seen with weight reduction, rather than vice versa. Presence of HCM might also have caused diastolic dysfunction although, in such cases, improvement in diastolic function would also not be expected, making obesity the more likely cause. Considering that HCM is common in cats [14] and that we aimed to assess wall thickness, a HCM phenotype (i.e., increased LVWT) which was not clinically relevant at the time of the study was not an exclusion criterion.

A further limitation is that the changes in LVWT, although statistically significant with a medium-to-large effect size, could be the result of inter-observer and intra-observer variability or daily variation. A control group to monitor for change in wall thickness over time in cats with a stable weight, would have helped eliminate this possibility. However, given that the differences documented were greater than the expected variation of LV wall measurements (both inter-observer and intra-observer coefficients of variation ≤ 5.9% for LV wall measurements by this group) [27] the changes observed are more likely to reflect genuine changes. The clinical significance of small changes in LVWT could be questioned; not least given that other factors (such as hydration status) might instead be responsible. However, this is likely to be an inherent issue of measuring wall thickness in cats. The changes reported in this study of 0.5–0.85 mm, although small, are clinically relevant when considering such a change could be the difference between a diagnosis of normal or a HCM phenotype. Finally, study investigators could not be blinded as to whether a cat had achieved target weight loss, as only such cats underwent repeat echocardiography; however, they were blinded to the baseline echocardiography results at time of repeat echocardiography.

## Conclusions

An increase in LVWT and diastolic dysfunction were common echocardiographic features in cats with obesity within our study and may be reversible with successful weight reduction. This highlights the importance of weight management in these cats. Further research is needed to investigate the clinical importance of these echocardiographic changes in otherwise healthy cats, and in cats with HCM.

## Methods

### Study animals

Cats referred to the Royal Canin Weight Management Clinic, University of Liverpool, for assessment and management of obesity, were recruited between August 2016 and November 2018. To meet eligibility criteria, cats could not have had significant cardiac or systemic disease, as assessed during initial examinations (as below). To be included in the final assessment, cats had to have reached their weight reduction target by the study end date (November 2019). The study protocol was approved by the University of Liverpool Veterinary Research Ethics Committee (RETH000353 and VREC793) and the Royal Canin Ethical Review Committee (150,720–55). Owners of all participating animals gave written, informed consent.

### Weight reduction regimen

Details of the weight reduction protocol used have been previously described [40, 41]. In brief, cats were determined to be clinically well with no systemic disease that may affect the ability to lose weight, based on physical examination, haematology, serum biochemistry, urinalysis and free thyroxine, performed during the initial visit. Body condition score was assessed using a nine-point scale [42]. DEXA was performed under sedation (after cardiac evaluation as follows) with midazolam (0.2 mg/kg), fentanyl (5 mcg/kg) and medetomidine (15 mcg/kg) intramuscular, reversed with atipamezole (37.5 mcg/kg) intramuscular. Body composition was analysed by DEXA, and data used to create individualised weight reduction plans for each cat, establishing both an ideal and a target weight, as previously described [40, 43]. In brief, the ideal weight was defined as the weight at which the cat’s body fat mass was optimal, based on DEXA body composition data. Target weight was the goal weight set for the period of controlled weight reduction. In most cases, target weight matched ideal weight; however, in some cases, a partial weight reduction protocol was used whereby the target weight set was greater than the ideal weight, with decisions made based on age and severity of obesity [44]. In such cases, the aim was for the cat to lose enough weight to lead to improved health and wellbeing, even though the cat would still be in overweight condition at the end of their period of weight reduction.

Commercially available, wet and dry therapeutic diets (Supplementary Table 3) were used for the controlled weight reduction protocol, with the choice of formulation dependent on owner and cat preferences. Food allocation was estimated by calculating the metabolic energy requirement based on the ideal weight, as previously described [40]. Individualised advice on lifestyle and activity alterations were also given to assist in weight reduction by a registered veterinary nurse (GRTW).

Cats were reweighed every two to four weeks to assess progress, with subsequent changes to the weight reduction diet if required. Cats were deemed to have reached the primary endpoint if target weight was achieved within the study period. Full laboratory analysis and DEXA were repeated at time of achieving target weight.

### Cardiovascular evaluation

Cardiac evaluation was performed prior to sedation for DEXA at both the initial visit and after reaching target weight. Cats considered to have a mild, preclinical HCM phenotype (stage B1 [20]) were not excluded based on the aim of assessing LVWT. Presence of congenital heart disease or cardiomyopathies other than HCM were considered exclusion criteria.

### Systolic blood pressure

Systolic blood pressure was measured indirectly by the Doppler method (Ultrasonographic Doppler Flow Detector 811-B; Parks Medical Electronics), as previously described [45], by an experienced operator (cardiology nurse, GRTW, or the echocardiographer). SBP was measured in a quiet room with gentle handling prior to other procedures. Cats were allowed to acclimatise to the environment before five measurements were taken, with the mean value recorded. Values equal to and exceeding 160 mmHg were considered consistent with hypertension [45].

### Cardiac biomarkers

Blood was collected into EDTA tubes by jugular venepuncture at the initial and last assessments. Samples were immediately centrifuged and separated EDTA-plasma stored at -20 °C until after study completion and sent as a single batch on dry ice to an external laboratory (IDEXX Laboratories, Wetherby, West Yorkshire, UK) for measurement of high sensitivity cardiac troponin I (Beckman Coulter Access hs-cTnI assay; IDEXX Laboratories) and second-generation N-terminal Pro B-type natriuretic peptide (Cardiopet proBNP test, IDEXX Laboratories).

### Electrocardiography

Six-lead ECG was obtained from all cats restrained in right lateral recumbency or sternal (dependent on temperament). Routine analysis of the ECG was performed, including rate, rhythm and standard lead II measurements. To assess HRV, the R-R interval for 20 consecutive cardiac cycles was measured. The VVTI was then calculated as the natural logarithm of the variance of these R-R intervals (VVTI = Ln[SD_RR_]^2^) [46].

### Echocardiography

Complete 2D, M-mode, colour flow, spectral and tissue Doppler echocardiography was performed with a Vivid 7 ultrasound machine (GE Healthcare), using a 10 MHz transducer, by an EBVS® European Veterinary Specialist in Small Animal Cardiology or a resident in training under the direct supervision of such a specialist. Echocardiography was performed without sedation, with cats positioned in both right and left lateral recumbency. Vagal manoeuvres (nasal planum pressure [47]) were utilised to attempt to separate E and A waves when summation occurred. Simultaneous ECG was used for timing of events during the cardiac cycle. Analysis was performed on a remote, off-line measuring system,^1^ by the operator who performed the echocardiography. The mean value of three cardiac cycles, in sinus rhythm was obtained for each variable and used in analysis.

Left atrial diameter (LADmax) and short axis left atrium to aorta ratio (LA/Ao) were measured as previously described [48–50]. LA enlargement was defined as LADmax ≥ 16 mm and/or LA/Ao ≥ 1.6. Measurement of end-diastolic interventricular septum (IVSd) and left ventricular free wall (LVFWd) thickness was made on 2D echocardiography as previously described [51], utilising a leading-edge-to-trailing edge method for measurement of the septum, leading-edge-to-leading edge for the free wall. The IVSd was measured in basal, mid and apical regions and the LVFWd measured in basal and mid regions (on three cardiac cycles and the mean for each region calculated), ensuring inclusion of any regions of focal hypertrophy, but exclusion of points of false tendon attachment or endocardial thickening; the region of maximal thickness for each wall was defined as the maximal IVSd and maximal LVFWd and utilised in statistical analysis. An increase in LVWT, which would be considered consistent with a HCM phenotype, was defined by a maximal diastolic wall thickness ≥ 6 mm; values of 5.5 to 5.9 mm were considered equivocal, and values < 5.5 mm were considered normal [20]. M-mode of the LV was obtained from a right parasternal short axis view at the level of the chordae tendineae, with the cursor bisecting the LV cavity symmetrically. M-mode fractional shortening was calculated using the standard formula [51]. Cats were classified as having either normal left ventricular diastolic function, impaired relaxation or pseudonormal diastolic function based on combined assessment of transmitral flow, measurement of E wave and A wave velocities and velocity ratios, IVRT duration and pw-TDI acquired as previously described [51, 52]. Transmitral data of cats with complete mitral E and A wave summation were excluded; partial summation was allowed if the A wave started late in E deceleration (when E wave was < 0.2 m/s) [53]. Pw-TDI was utilised to assess longitudinal motion of the septal and lateral mitral annuli and lateral tricuspid annuli (diastolic E’ and A’ and systolic S’ velocities; E’/A’ ratio) as previously described [52, 54, 55]. Cats with summated E’/A’ underwent vagal manoeuvres [47] to attempt to transiently separate them, but data was excluded if persistent E’A’ summation was present. Cats were classified as having impaired left ventricular relaxation based on one or more of the following criteria: a decrease in mitral E/A (< 1), pw-TDI LVFW E’A’ (< 1) or increase in IVRT (> 60 ms). If the transmitral E/A and/or IVRT were within reference ranges but pw-TDI LVFW E’A’ < 1, this was considered pseudonormal diastolic function [51, 52]. For cats that reached the end point, cardiac evaluations were repeated, allowing comparison between the two time-points.

### Statistics

Statistical analysis was performed with the use of commercially available software (SPSS 28.0). Sample size was based on pragmatic recruitment within the study time frame. For every cat, a mean of each echocardiographic and clinical variable was recorded for each time-point. On account of the small sample size, non-parametric tests were used. The median (and IQR) was reported for all descriptive statistics. Baseline weight, age and BCS were compared between the cats that did and did not achieve weight reduction using a Mann–Whitney U test. For the cats that completed the study, a Wilcoxon-signed rank test was used to compare continuous variables pre- and post- weight reduction and a marginal homogeneity test used to compare categorical variables (diastolic function class). For both the Mann–Whitney U and Wilcoxon-signed ranks test, effect size was calculated from the z statistic, and used to calculate *r*, as described by Cohen [19], using the formula *r* = Z / √N (where Z is the z statistic and N is the sample size). The effect size, *r*, was interpreted according to the rules of Cohen [19], whereby values of 0.1, 0.3 and ≥ 0.5 were considered small, medium and large effects respectively [18, 19]. The level of statistical significance was set at *P* < 0.05, for two-sided analyses.

### Supplementary Information

**Additional file 1: Supplementary Table 1.** Baseline demographic and cardiovascular data for all enrolled cats. Baseline data for all 20 enrolled cats (sex, age, body weight, body condition score, blood pressure, heart rate, electrocardiographic and echocardiographic diagnosis).

**Additional file 2: Supplementary Table 2.** Epidemiological and cardiovascular variables at baseline for all 20 cats. Baseline epidemiological and cardiovascular variables for all 20 cats given as median and interquartile range.

**Additional file 3: Supplementary Table 3.** Average composition of the therapeutic diets used for weight reduction. Composition of the two commercially available weight management diets used in the weight management regimen.

## Data Availability

All data generated or analysed during this study are included in this published article [and its supplementary information files].

## Abbreviations

2D: Two dimensional
BCS: Body condition score
DEXA: Dual energy X-ray absorptiometry
ECG: Electrocardiography
HCM: Hypertrophic cardiomyopathy
HRV: Heart rate variability
Hs-cTnI: High-sensitivity cardiac troponin I
IQR: Interquartile range
IVRT: Isovolumetric relaxation time
IVSd: End-diastolic interventricular septum thickness
LA: Left atrium
LA/Ao: Short axis left atrium to aorta ratio
LADmax: Maximal left atrial diameter
LV: Left ventricle / left ventricular
LVFWd: End-diastolic left ventricular free wall thickness
LVWT: Left ventricular wall thickness
LVOT: Left ventricular outflow tract
NT-proBNP: N-terminal Pro B-type Natriuretic Peptide
PW-TDI: Pulsed wave tissue Doppler imaging
RI: Reference interval
SBP: Systolic blood pressure
VVTI: Vasovagal tonus index

## Footnotes

1 GE Echopac version 113, GE Medical Systems, Buckinghamshire, UK.
