# Supplementary material for: The effect of obesity and subsequent weight reduction on cardiac morphology and function in cats
Source: BMC Vet Res. 2024 Apr 24;20:154. doi: 10.1186/s12917-024-04011-0 (PMC11040875; doi:10.1186/s12917-024-04011-0)
Supplement: Supplementary file 1 — Additional file 1: Supplementary Table 1. Baseline demographic and cardiovascular data for all enrolled cats. Baseline data for all 20 enrolled cats (sex, age, body weight, body condition score, blood pressure, heart rate, electrocardiographic and echocardiographic diagnosis). [file 12917_2024_4011_MOESM1_ESM.docx]

**Supplementary table 1: Baseline demographic and cardiovascular data for all enrolled cats.**

| **Cat** | **Breed** | **Sex** | **Age (years/months)** | **BW (kg)** | **BCS (/9)** | **Weight reduction achieved (Yes/no)** | **SBP (mmHg)** | **ECG HR (bpm)** | **ECG findings** | **Echocardiographic diagnosis** | **Presence of LVOTO (yes/no)** |
| --- | --- | --- | --- | --- | --- | --- | --- | --- | --- | --- | --- |
| 1 | DSH | MN | 6y 6m | 6.5 | 8 | Yes | 100 | 200 | Sinus rhythm | Normal | No |
| 2 | DSH | FN | 7y 3m | 5.5 | 8 | Yes | 150 | 160 | Sinus rhythm | Focal HCM phenotype (IVS bulge) | No |
| 3 | DSH | MN | 10y 1m | 9.05 | 9 | Yes | 140 | 180 | Sinus rhythm | HCM phenotype | No |
| 4 | DSH | MN | 8y 0m | 6.35 | 8 | Yes | ND | 140 | Sinus rhythm | HCM phenotype | No |
| 5 | DSH | MN | 1y 3m | 6.4 | 8 | Yes | 104 | 180 | Sinus rhythm | Normal | No |
| 6 | DSH | MN | 12y 9m | 7.22 | 9 | Yes | 120 | 160 | Sinus rhythm, left anterior fascicular block | Equivocal HCM phenotype | No |
| 7 | DSH | MN | 1y 7m | 6.57 | 8 | Yes | 120 | 160 | Sinus rhythm, intraventricular conduction disturbance | Focal HCM phenotype (IVS bulge) | No |
| 8 | DSH | FN | 9y 3m | 7.32 | 9 | Yes | 128 | 160 | Sinus rhythm | Equivocal HCM phenotype | Yes- Mild |
| 9 | DSH | MN | 7y 5m | 10.5 | 9 | Yes | 150 | 220 | Sinus rhythm | HCM phenotype | No |
| 10 | DSH | MN | 8y 1m | 7.2 | 7 | Yes | 144 | 230 | Sinus rhythm Interventricular conduction disturbance | HCM phenotype | No |
| 11 | DSH | MN | 8y 2m | 9.11 | 9 | Yes | 150 | 180 | Sinus rhythm | HCM phenotype | No |
| 12 | DSH | FN | 5y 2m | 9.75 | 9 | No | 130 | 180 | Sinus rhythm | HCM phenotype | No |
| 13 | DSH | MN | 2y 1m | 8.3 | 9 | No | ND | 160 | Sinus rhythm | HCM phenotype | No |
| 14 | DSH | MN | 9y 1m | 6.44 | 7 | No | 125 | 120 | Sinus bradycardia | Normal | No |
| 15 | DSH | FN | 11y 3m | 7.2 | 9 | No | 150 | 220 | Sinus rhythm | HCM phenotype | No |
| 16 | DSH | FN | 7y 6m | 8.4 | 9 | No | 120 | 240 | Sinus rhythm | Equivocal HCM phenotype | No |
| 17 | DSH | FN | 5y 7m | 7.1 | 9 | No | 145 | 160 | Sinus rhythm | Equivocal HCM phenotype | No |
| 18 | DSH | FN | 6y 5m | 6.88 | 9 | No | 140 | 220 | Sinus rhythm | HCM phenotype with LVOTO. | Yes |
| 19 | DSH | MN | 8y 6m | 7.76 | 9 | No | 110 | 160 | Sinus rhythm | Equivocal HCM phenotype | No |
| 20 | DSH | FN | 5y 8m | 4.99 | 8 | No | 146 | 180 | Sinus rhythm | Normal | No |

BCS: body condition score, BW: body weight, DSH: domestic shorthair, ECG: electrocardiography, FN: female neutered, HCM: hypertrophic cardiomyopathy, HR: heart rate, IVS: interventricular septum, LVOTO: left ventricular outflow tract obstruction, MN: male neutered, ND: not done, SBP: blood pressure
