# Supplementary material for: The effect of obesity and subsequent weight reduction on cardiac morphology and function in cats
Source: BMC Vet Res. 2024 Apr 24;20:154. doi: 10.1186/s12917-024-04011-0 (PMC11040875; doi:10.1186/s12917-024-04011-0)
Supplement: Supplementary file 2 — Additional file 2: Supplementary Table 2. Epidemiological and cardiovascular variables at baseline for all 20 cats. Baseline epidemiological and cardiovascular variables for all 20 cats given as median and interquartile range. [file 12917_2024_4011_MOESM2_ESM.docx]

**Supplementary Table 2:** **epidemiological and** **cardiovascular variables at baseline for all 20 cats.**

| **Variable** | **Median** | **(IQR)** | **Reference interval** |
| --- | --- | --- | --- |
| **Age (years/months)** | 7y5m | 5y6m-8y11m |  |
| **Body weight (kg)** | 7.20 | 6.46-8.38 |  |
| **BCS (/9)** | 8 | 8-9 |  |
| **ECG Heart rate (bpm)** | 180 | 160-215 | 140-240 |
| **Heart rate variability (VVTI)** | 5.21 | 4.93-5.70 | 3.64-5.64 |
| **SBP (mmHg)** | 135 | 120-147 | <160 |
| **Hs-cTnI (ng/mL)** | 0.007 | 0.004-0.038 | <0.04 |
| **NT-proBNP (pmol/L)** | 23.9 | 23.9-27.5 | <100 |
| **LA/Ao** | 1.30 | 1.22-1.44 | <1.6 |
| **LADmax (mm)** | 15.1 | 13.7-16.0 | <16.0 |
| **Max-IVSd (mm)** | 6.05 | 5.23-6.58 | < 6.0 |
| **Max-LVFWd (mm)** | 5.55 | 5.13-5.98 | <6.0 |
| **Mitral E velocity (m/s)** | 0.65 | 0.57-0.70 |  |
| **Mitral E/A** | 1.02 | 0.85-1.22 | 1-2 |
| **IVRT (ms)** | 59.0 | 54.0-63.0 | 37-60 |
| **Mitral septal (IVS) E’/A’** | 0.60 | 0.55-0.81 | 1-2 |
| **Mitral lateral (LVFW) E’/A’** | 0.67 | 0.57-0.95 | 1-2 |
| **Right lateral E’/A’** | 0.64 | 0.56-0.75 | 1-2 |
| **Septal S’ (cm/s)** | 6.5 | 4.9-9.0 | >6 |
| **Lateral S’ (cm/s)** | 6.6 | 4.8-9.4 | >6 |
| **Fractional shortening (%)** | 54.1 | 48.3-59.5 | >30 |

BCS: body condition score, ECG: electrocardiography, hs-cTnI: high sensitivity cardiac troponin I, IQR: interquartile range, IVRT: isovolumetric relaxation time, IVSd end-diastolic interventricular septum thickness, LA/Ao left atrium: aorta ratio, LADmax: maximal left atrial diameter, LVFWd: end-diastolic left ventricular free wall thickness, NT-proBNP: N-type N-terminal pro-brain natriuretic peptide, SBP: systolic blood pressure, VVTI: vasovagal tonus index
