# Supplementary material for: The effect of obesity and subsequent weight reduction on cardiac morphology and function in cats
Source: BMC Vet Res. 2024 Apr 24;20:154. doi: 10.1186/s12917-024-04011-0 (PMC11040875; doi:10.1186/s12917-024-04011-0)
Supplement: Supplementary file 3 — Additional file 3: Supplementary Table 3. Average composition of the therapeutic diets used for weight reduction. Composition of the two commercially available weight management diets used in the weight management regimen. [file 12917_2024_4011_MOESM3_ESM.docx]

**Supplementary Table 3: Average composition of the therapeutic diets used for weight reduction.**

|  | **High protein high fibre dry ^a^** | | **High protein wet ^b^** | |
| --- | --- | --- | --- | --- |
| **ME content (kcal/kg)** | 3113 | | 640 | |
|  | Per 100g AF | g/1000 kcal ME | Per 100g AF | g/1000 kcal ME |
| **Moisture** | 5.5 | 18 | 84.0 | 1313 |
| **Protein** | 34.0 | 111 | 7.5 | 117 |
| **Crude fat** | 9.0 | 29 | 2.2 | 31 |
| **Digestible carbohydrate ^c^** | 19.1 | 62 | 3.1 | 48 |
| **Crude fibre** | 13.9 | 45 | 1.5 | 23 |
| **Total dietary fibre** | 23.6 | 77 | 1.5 | 23 |
| **Ash** | 8.8 | 29 | 1.9 | 30 |

^a^ High protein high fibre dry food (Satiety Weight Management, Royal Canin); ^b^ high protein wet food (Obesity Management, Royal Canin); ^c^ digestible carbohydrate fraction (e.g. sugars and starch) calculated using the following predictive equation: digestible carbohydrate [g] = dry matter [g] - (crude fat [g] + crude protein [g] + ash [g] + total dietary fibre [g]). AF: as fed; DM: dry matter; ME: metabolisable energy content, calculated using a predictive equation based on total dietary fibre.
